# Exosomes derived from human adipose mesenchymal stem cells improve ovary function of premature ovarian insufficiency by targeting SMAD

**DOI:** 10.1186/s13287-018-0953-7

**Published:** 2018-08-09

**Authors:** Boxian Huang, Jiafeng Lu, Chenyue Ding, Qinyan Zou, Wei Wang, Hong Li

**Affiliations:** 1 Center of Reproduction and Genetics, Affiliated Suzhou Hospital of Nanjing Medical University, Suzhou Municipal Hospital, Suzhou, 215002 China; 2 Central Laboratory, Affiliated Suzhou Hospital of Nanjing Medical University, Suzhou Municipal Hospital, Suzhou, 215002 China; 3 State Key Laboratory of Reproductive Medicine, Nanjing Medical University, Nanjing, 210029 China

**Keywords:** Human adipose stem cells, Premature ovarian insufficiency, Exosome, SMAD pathway

## Abstract

**Background:**

Although many reports show that various kinds of stem cells have the ability to recover the function of premature ovarian insufficiency (POI), few studies are associated with the mechanism of stem cell treatment of POI. We designed this experimental study to investigate whether human adipose stem cell-derived exosomes (hADSC-Exos) retain the ability to restore ovarian function and how hADSC-Exos work in this process.

**Methods:**

A POI mouse model was established and human ovarian granule cells (hGCs) collected from individuals with POI were prepared to assess the therapeutic effects and illuminate the mechanism of hADSCs in curing POI. The hematoxylin and eosin assay method was employed to assess the number of follicles. Enzyme-linked immunosorbent assay (ELISA) was used to detect the serum levels of sex hormones. The proliferation rate and marker expression levels of hGCs were measured by flow cytometry (fluorescence-activated cell sorting). Real-time PCR and western blot assays were used to determine the mRNA and protein expression levels of SMAD2, SMAD3, and SMAD5. Western blot assays were used to test the protein expression levels of apoptosis genes (Fas, FasL, caspase-3, and caspase-8).

**Results:**

After the hADSC-Exos were transplanted into the POI mice model, they exerted better therapeutic activity on mouse ovarian function, improving follicle numbers during four stages. ELISA results showed that hADSC-Exos elevated the hormone levels to the normal levels. In addition, after hADSC-Exos were cocultured with POI hGCs, our results showed that hADSC-Exos significantly promoted the proliferation rate and inhibited the apoptosis rate. Furthermore, hADSC-Exos also increased the marker expression of hGCs to the normal level. Besides, mRNA and protein assays demonstrated that hADSC-Exos downregulated the expression of SMAD2, SMAD3, and SMAD5 in vivo and in vitro. Western blot assay demonstrated that hADSC-Exos inhibited expression of the apoptosis genes in POI hGCs, and SMAD knockdown increased the protein expression of apoptosis genes.

**Conclusions:**

These findings demonstrate for the first time the molecular cascade and related cell biology events involved in the mechanism by which exosomes derived from hADSCs improved ovarian function of POI disease via regulation of the SMAD signaling pathway.

**Electronic supplementary material:**

The online version of this article (10.1186/s13287-018-0953-7) contains supplementary material, which is available to authorized users.

## Background

Premature ovarian insufficiency (POI), also termed premature ovarian failure (POF), is the cessation of ovarian function before the age of 40 years. It is characterized by amenorrhea, hypoestrogenism, hypergonadotropism, infertility, and decreased number of follicles. It affects 1% of women by age 40 years and 0.1% by age 30 years [1]. Ovarian follicles have high susceptibility to apoptosis induced by chemotherapic drugs. Cancer-directed therapies can cause accelerated loss of ovarian reserve and atrophy [2]. Currently, POI cannot be reversed and although treatments are available using long-term hormone replacement therapy to relieve menopausal symptoms, it is hard to prevent premature ovarian aging in women [3]. There is therefore an urgent need to improve treatment strategies. Regenerative medicine research suggests that stem cell therapy is a promising measure in the treatment of various human diseases because of the stem cells’ self-renewal capacity and multiplex differentiation potential to replace the damaged tissue or their capacities of producing paracrine factors to rescue injured tissues [4, 5].

Human adipose mesenchymal stem cells (hADSCs) are derived from human adipose tissue as a kind of superior biomaterial that can be suitable for allotransplantation and regenerative medicine [6]. There are several advantages which make hADSCs suitable for clinical therapy: they stably expand under basic culture conditions, they exhibit a low risk of contamination, and they have low immune rejection following transplantation [7]. Meanwhile, compared to bone marrow mesenchymal stem cells (BMSCs), deriving stem cells from human adipose tissue is a simple and minimally invasive procedure [8].

Exosomes are endosomal-origin, small-membrane vesicles secreted by a variety of stem cells after invagination of the plasma membrane and then released into the extracellular space, sizes ranging from 40 to 100 nm in diameter [9]. The exosomes are involved in cell-to-cell communication and have the ability to regulate the fate and phenotype of recipient cells by delivering microRNAs, mRNAs, and active proteins [10]. Recently, accumulating evidence has indicated that exosomes derived from stem cells transfer proteins and RNA from cell to cell, rationalizing potential applications to cure many diseases, such as hepatic failure, heart failure, and aging [11–13]. However, the effects of hADSC exosomes (hADSC-Exos) on ovary damage and the underlying mechanisms are not well characterized.

SMA and mother against decapentaplegic (MAD)-related proteins (SMADs) are intracellular components of the TGF-β signaling pathway [14]. In mammalian species, SMAD effectors are required for germline establishment. SMADs consist of receptor-regulated SMADs (SMAD1/2/3/5/9) that play a key role in oogenesis and proliferation of granular cells [15]. The SMAD2/3 signaling pathway is involved in regulating expansion and steroidogenic activity of porcine oocyte–cumulus cell complex stimulated in vitro with either FSH or EGF [16]. Previous study results showed that SMAD2 and SMAD3 function redundantly to maintain female fertility by regulating follicular development, ovulation, and cumulus cell expansion, and SMAD3-knockout mice demonstrate ovulatory defects and lack corpora lutea in the ovary [17]. SMAD5 is indispensable for primordial germ cell proliferation. Knocking down SMAD5 expression increased the rate of apoptosis, as well as the levels of Fas, FasL, caspase-8, and caspase-3 protein in germ cells [18]. Furthermore, SMAD5 knockout mice have been shown to die in the uterus during embryogenesis, which shows defective primordial germ cell formation and deficient mouse embryos [19].

In the present study, we investigated the therapeutic effects of hADSC-Exos in a POI mouse model. Consistent with our hypothesis, we found that hADSC-Exos could significantly attenuate ovary damage. We further observed a remarkable follicle number increase and hormone level recovery in the POI mouse model, which indicated that hADSC-Exos may be one way to alleviate POI. An in-vitro study demonstrated that hADSC-Exos can promote the proliferation rate and inhibit the apoptosis rate in POI hGCs. Furthermore, hADSC-Exos can increase mRNA and protein expression of SMAD2, SMAD3, and SMAD5 in vivo and in vitro. Meanwhile, hADSC-Exos inhibit the expression of Fas/FasL, caspase-3, and caspase-8 by activating SMAD. To the best of our knowledge, this is the first study to suggest that hADSC-Exos exhibit a recovery function for POI, which indicates that hADSC-Exos can become a novel regulator in ovary damage therapy.

## Methods

### Preparation and culture of hADSCs

As described previously, hADSC lines were established in our laboratory [4]. The cells were planted in a 10-cm^2^ culture dish at a density of 1 × 10^7^ cells in 10 ml of regular growth medium [4] and were incubated at 37 °C and 5% CO_2_. All of the experiments were performed with third or fourth-passage hADSCs.

### Establishment of POI mouse model

Female ICR mice between 7 and 8 weeks of age were obtained from Nanjing Medical University with Institutional Animal Care and Use Committee approval in accordance with institutional guidelines. According to our previous method, the cyclophosphamide (CTX, 120 mg/kg, 2 weeks) treatment method was employed to build the POI mouse model [1]. The animals were divided into four groups (*n* = 10 per group) after CTX treatment: control group (noninjection), CTX treatment with PBS group, CTX treatment with hADSCs group and CTX treatment with hADSC-Exos group. Follicle numbers and hormone levels were estimated after cell transplantation. They were bred at a temperature of 28 ± 2 °C with a 12-h light/dark cycle. Vaginal smears of the mice were taken to determine the estrous cycle at 9:00 a.m. daily.

### Isolation of primary hGCs from POI patients

Subjects (age < 40) with tubal occlusion were used as the control group. POI patients were recruited according to the following inclusion criteria: primary amenorrhea or secondary amenorrhea for at least 4 months; age < 40 years; and at least two records of FSH measurements with FSH serum concentrations exceeding 40 mIU/ml. Women with a known normal karyotype, previous radiotherapy, autoimmune diseases, or ovarian surgery were excluded. Primary hGCs were obtained after informed consent was provided by the normal subjects with tubal occlusion (*n* = 34) and POI patients (*n* = 41) after approval from the Suzhou Hospital Affiliated to Nanjing Medical University Research Ethics Board. All subjects were treated with recombinant FSH (Puregon; Schering-Plough, NJ, USA) and GnRH antagonist Ganirelix (Merck, Frosst, Montreal, Canada). Vaginal ultrasound examinations were performed to monitor follicular development. Final follicular maturation was induced by administering 10,000 IU of human chorionic gonadotropin (hCG) (Pregnyl; Merck). hGCs were purified using density centrifugation from follicular aspirates collected from women undergoing oocyte retrieval as described previously [20]. Primary hGCs were cultured in six-well plates in DMEM/F12 media (Thermo, USA) containing 1% penicillin/streptomycin, 10% fetal bovine serum (complete medium), 100 mg/ml streptomycin sulfate (Thermo, USA), and 1× GlutaMAX (Thermo, USA). The culture medium was changed every other day in all of the experiments.

### Isolation of hADSC-derived exosomes

Exosomes were collected from conditioned medium (CM) of hADSCs using the ExoQuick-TC (SBI, Mountain View, CA, USA). Briefly, hADSCs at approximately 80% confluence were cultured for 48 h and the CM was collected, centrifuged at 2000 × *g* for 20 min and filtrated through 0.22-μm filters (Millipore, Billerica, MA, USA) to remove cell debris. Then 10 ml of supernatant was mixed with 2 ml of ExoQuick precipitation solution and the mixture incubated overnight at 4 °C. After incubation, exosomes were centrifuged at 1500 × *g* for 30 min to form a pellet, which aspirated the supernatant, and spun down at 1500 × *g* for 5 min to remove residual ExoQuick-TC. The exosome pellet was resuspended in 100 μl phosphate buffered saline (PBS). The exosome-enriched fraction was diluted with 100 μl PBS and stored at − 80 °C. The protein content of the concentrated exosomes was determined using a BCA protein assay kit (Thermo Fisher, USA). The hADSCs-Exos were confirmed to express the exosome markers (CD63, CD9, and CD81) using a flow cytometry (FACS) method and western blot analysis.

### Electron microscopy analysis of hADSC-derived exosomes

The exosome pellet derived from hADSCs was diluted to 1 mg/ml with PBS. Then, a specimen of exosomes was spotted onto a glow-discharged copper grid on the filter paper and dried for 20 min under an infrared lamp, Finally, the grid was stained with 3% (w/v) phosphotungstic acid (Electron Microscopy Sciences, Washington, PA, USA) and air-dried at room temperature, and the exosomes were examined under transmission electron microscopy with an accelerating voltage of 80 kV (H-600; Hitachi, Tokyo, Japan).

### hADSC-Exos were cocultured with hGCs and injected into the ovaries of POI mice

Some of the aforementioned hGCs were divided into the exosome cocultured group and the PBS cocultured control group. After 7 days, hGCs were used for study. The POI mice model was divided into the exosome-injected group (an approximate amount produced by 1 × 10^6^ cells), the hADSC-injected group, the PBS-injected group, and a noninjected control group. After 4 weeks, the mice in each group were killed to evaluate the follicle numbers by hematoxylin and eosin (HE) assay and the hormone level by ELISA.

### Assessment of ovarian function by a comparison of the ovarian follicle counts

After cell transplantation, the mice (cell transplanted and nontransplanted) were euthanized from 0 to 4 weeks; the ovaries on both sides were removed and fixed by 10% formalin, paraffin embedded, serially sectioned with a thickness of 5 mm, mounted in order on glass microscope slides, and stained with HE. Four stages of follicles (primordial, primary, secondary, and antral follicles) were detected and classified. The ratio of the number of follicles from the ovary was calculated and compared between each group (*n* = 10 per group). Three representative sections from each ovary were selected. Only follicles containing an oocyte were counted to avoid counting any follicle twice. The experiments were repeated three times, and the results are presented as the fold change ± SD. *p* < 0.05 determined a significant difference.

### ELISA analysis

Plasma from the POI mice model was harvested after exosome injection to evaluate the expression level of E2, AMH, or FSH using an ELISA kit (Mybiosource, USA) according to the guidelines of the manufacturer. Briefly, 50 μl of the serum sample was added per well. The test plate was wrapped with a membrane and incubated for 30 min at 37 °C. Thereafter, the wells on the plate were dried and washed five times with wash buffer (10 s per time). Then, 50 μl of the HRP-conjugate reagent was added into each sample well and incubated for 60 min at 37 °C. The samples were washed five times with wash buffer (10 s per time). Subsequently, 50 μl of substrate A solution followed by 50 μl of substrate B solution were added and incubated for 15 min at 37 °C. Then, 50 μl of stop solution was added into each control and the sample well. Finally, the light absorbance was measured and recorded by a spectrophotometer (Varian Company, Australia).

### FACS analysis

The hGCs were digested separately by trypsin–EDTA for 3 min and were blown into single cells gently, which were fixed and permeated by the Cytofix/Cytoper Fixation/Permeabilization Solution Kit (BD, USA) following the instructions of the manufacturer. The hGCs were then stained with PE or FITC-conjugated antibodies for anti-human-ki67 (Abcam, USA), anti-human-AMH (Thermo, USA), anti-human-FSHR (Thermo, USA), anti-human-FOXL2 (Thermo, USA), anti-human-CYP19A1 (Abgent, USA), anti-human-Annexin V (Abcam, USA), anti-human-CD9 (Abcam, USA), anti-human-CD63 (Abcam, USA), and anti-human-CD81 (Thermo, USA) or their corresponding isotype control for 30 min at 4 °C as already described. These stained cells were analyzed on a fluorescence-activated cell sorter (Beckman, USA). The experiments were repeated three times, and the results are presented as the fold change ± SD. *p* < 0.05 determined a significant difference.

### Gene silencing with RNA interference

SMAD2 siRNA (107873; Thermo Fisher), SMAD3 siRNA (107876; Thermo Fisher), and SMAD5 siRNA (115719; Thermo Fisher) were transfected into cells at final concentrations of 40 nM with the Dharmafect 1 (catalog number T-2001-02; Dharmacon) transfection reagent, following the manufacturer’s instructions, to silence SMAD2, SMAD3, and SMAD5 respectively in the hGCs. Cells were seeded onto 12-well plates before transfection so that the confluency reached 50% by the time of transfection. We used 2 μl of the transfection reagent, 2 μl of a 20-mM siRNA solution, and 4 × 10^4^ hGCs in 1 ml of culture medium. The efficiency of gene silencing was verified with western blot analysis and found to be optimal at 72 h.

### RNA extraction and real-time polymerase chain reaction

Total RNA from the hGCs from the POI patients, ovarian tissue from the POI mouse model, hGCs treated with PBS, exosomes, or hADSCs were extracted using the QIAGEN RNeasy Mini Kit (QIAGEN) and reverse-transcribed to cDNA with the PrimeScript RT Reagent Kit (Takara, Japan) according to the manufacturer’s methods. The total RNA concentration was determined by measuring the absorbance at 260 nm. Quantitative real-time polymerase chain reaction (PCR) was carried out with SYBR Premix Ex Taq (Takara, Japan) on a Thermal Cycler Dice Real Time System (Takara, Japan). Real-time PCR (ABI PRISM 7900; Applied Biosystems, USA) was carried out for 40 cycles of denaturation at 95 °C for 15 s, annealing at 58 °C for 15 s, and extension at 72 °C for 30 s. The cycle time (Ct) values were obtained after analysis with the Sequence Detection System and analysis software (Applied Biosystems). The 2^–ΔΔCt^ calculation method was used to analyze the data. Each sample was normalized to its GAPDH transcript content. Experiments were repeated three times. The results are presented as the fold change ± SD. *p* < 0.05 is considered a statistically significant difference. The primer sequences are presented in Additional file 1: Table S1.

### Western blot analysis

hGCs from the POI patients, ovarian tissues from the POI mouse model, hGCs treated with PBS, exosomes, or hADSCs were harvested and dissociated in a lysis buffer. Protein was extracted from each sample, which was then loaded onto 10% gels and separated by sodium dodecyl sulfate polyacrylamide gel electrophoresis (SDS-PAGE). Next, the separated proteins were transferred to polyvinylidene difluoride membranes (PVDF; Millipore, USA). The proteins were then incubated with the primary antibodies (Abcam, USA), namely, anti-human-SMAD2, anti-human-SMAD3, anti-human-SMAD4, anti-human-Fas, anti-human-FasL, anti-human-Caspase-3, anti-human-Caspase-8, anti-human-CD9, anti-human-CD63, anti-human-CD81, and anti-human-GAPDH, and subsequently incubated with the appropriate secondary antibodies (goat anti-rabbit HRP conjugates; Jackson Immunoresearch, West Grove, PA, USA). The specific signals were detected with enhanced chemiluminescence (Pierce ECL Western blotting Substrate; Thermo). Finally, the membrane was visualized with a chemiluminescence detection system (Tanon, China), and the signal intensity of each band was analyzed with ImageJ software (National Institutes of Health, USA). Experiments were repeated three times. The results are presented as the fold change ± SD. *p* < 0.05 is considered a statistically significant difference.

### Statistical analysis

All results are shown as the mean ± SD. Statistically significant differences were determined by one-way ANOVA with SPSS 17.0 software, and *p* < 0.05 was regarded as statistically significant.

## Results

### hADSC-Exos restored ovarian function in a POI mouse model

**Fig. 1:**
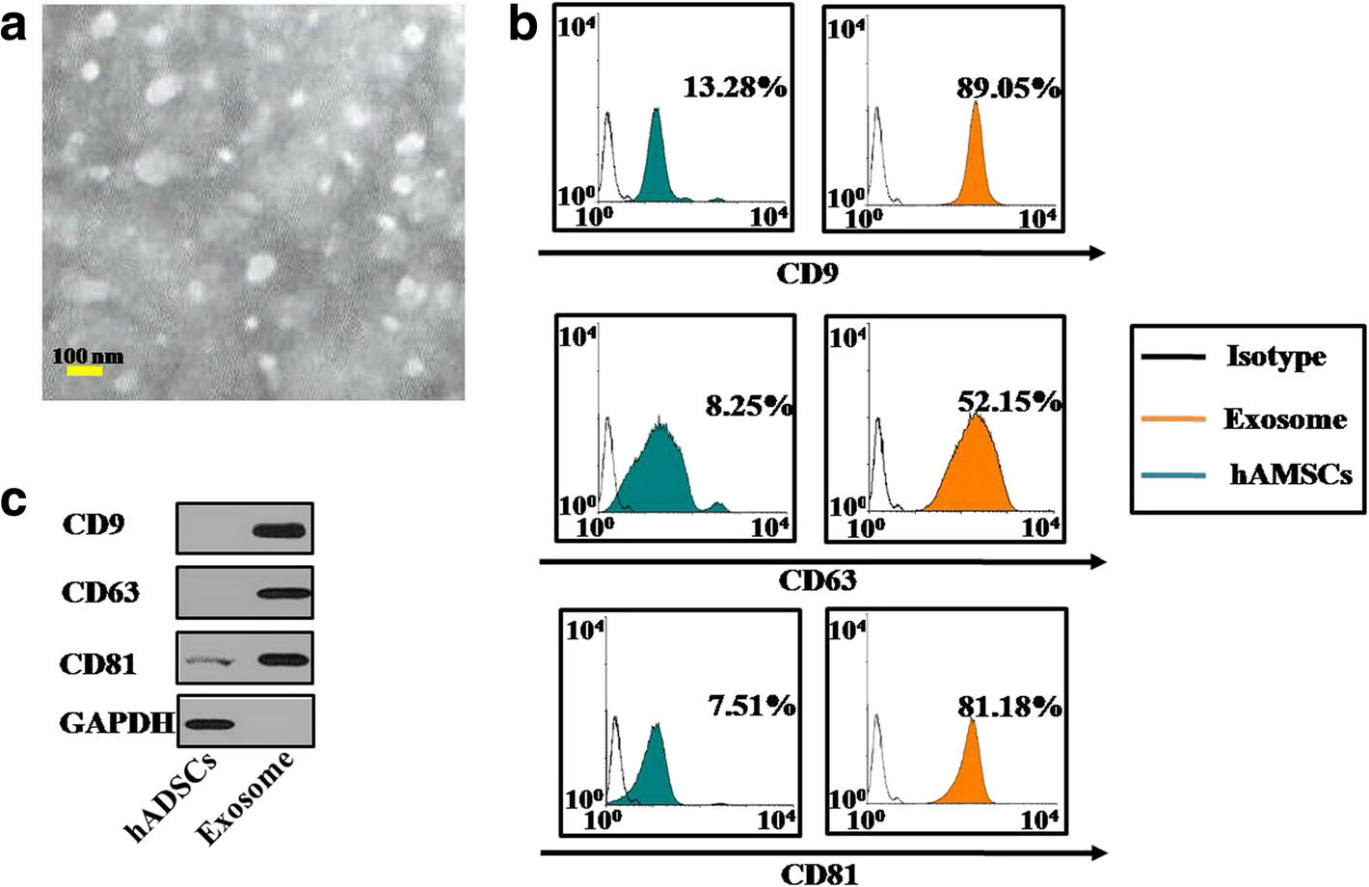
Characterization of hADSC-Exos. **a** Phenotype of hADSC-Exos detected by electron microscopy. **b** Expression levels of CD9, CD63, and CD81 in hADSC-Exos detected by flow cytometry. **c** Expression levels of CD9, CD63, and CD81 in hADSC-Exos detected by western blot. Scale bars = 100 nm. GAPDH glyceraldehyde 3-phosphate dehydrogenase, hADSC human adipose mesenchymal stem cells

**Fig. 2:**
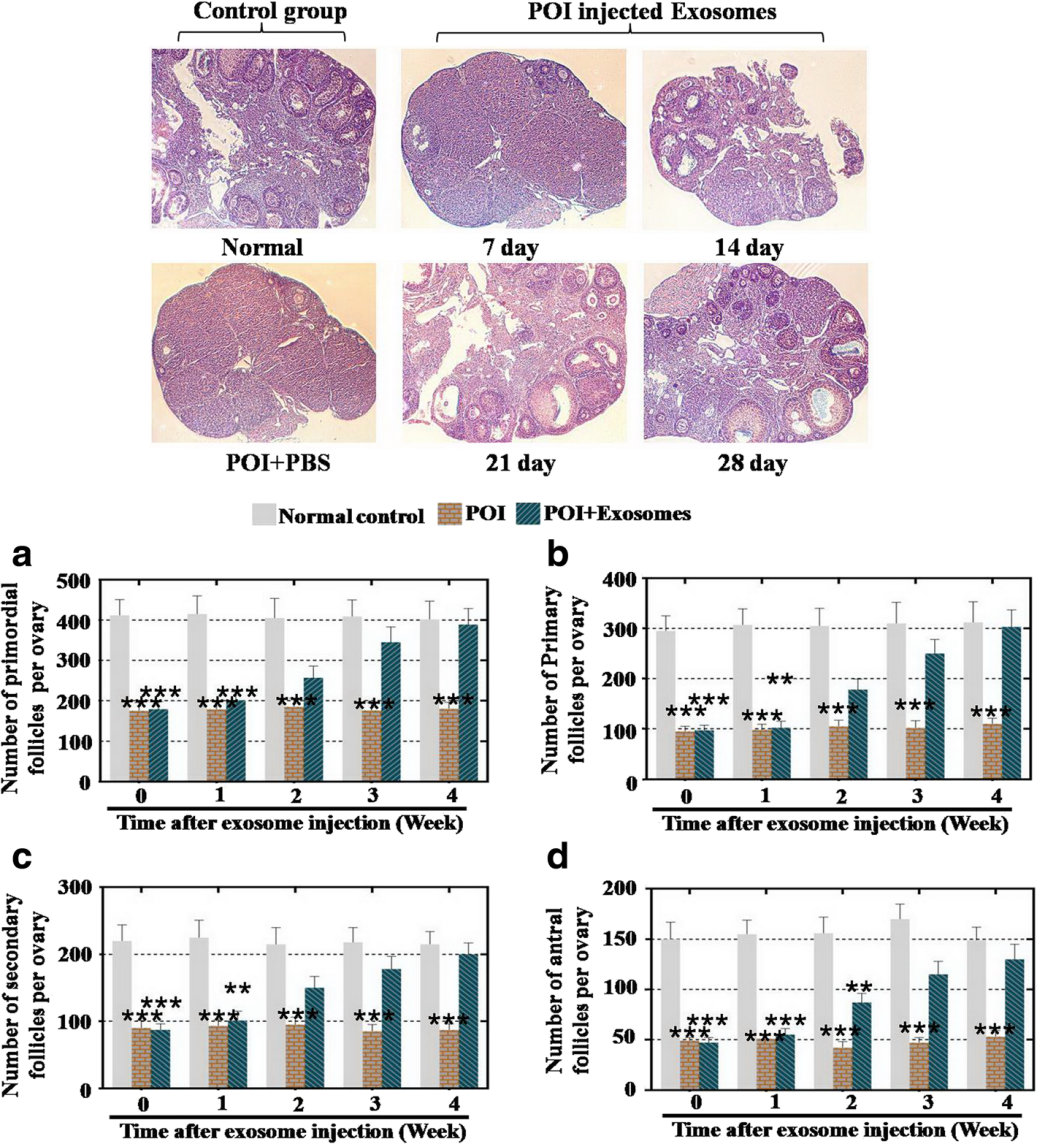
hADSC-Exos improved function of POI mouse model. **a** Number of primordial follicles counted over 4 weeks after hADSC-Exos injection. **b** Number of primary follicles counted over 4 weeks after hADSC-Exos injection. **c** Number of secondary follicles counted over 4 weeks after hADSC-Exos injection. **d** Number of antral follicles counted over 4 weeks after hADSC-Exos injection. All experiments carried three times; error bars indicate SD. **p < 0.01, ***p < 0.001 (compared with POI group). POI premature ovarian insufficiency, PBS phosphate buffered saline

First, to evaluate the effects of hADSC-Exos treatments on ovarian function in a POI mouse model, transmission electron microscopy (TEM) assay showed that hADSC-Exos displayed a round, ball-like shape and had diameters of approximately 40–100 nm (Fig. 1a). Fluorescence-activated cell sorting (FACS) was used to characterize the hADSC-Exos. Our results showed that the cell surface markers CD9, CD63, and CD81 in hADSC-Exos were more highly expressed than in hADSCs (Fig. 1b). Western blot assay was used to test the marker expression; our results revealed that the protein levels of CD9, CD63, and CD81 in hADSC-Exos were higher than in hADSCs (Fig. 1c). After hADSC-Exos were injected into the ovaries of POI mice, HE-stained ovarian tissues showed that hADSC-Exos could restore the follicle number to 96% primordial follicles, 97% primary follicles, 93% secondary follicles, and 87% antral follicles significantly at week 4 compared to the control group (Fig. 2a–d).

**Fig. 3:**
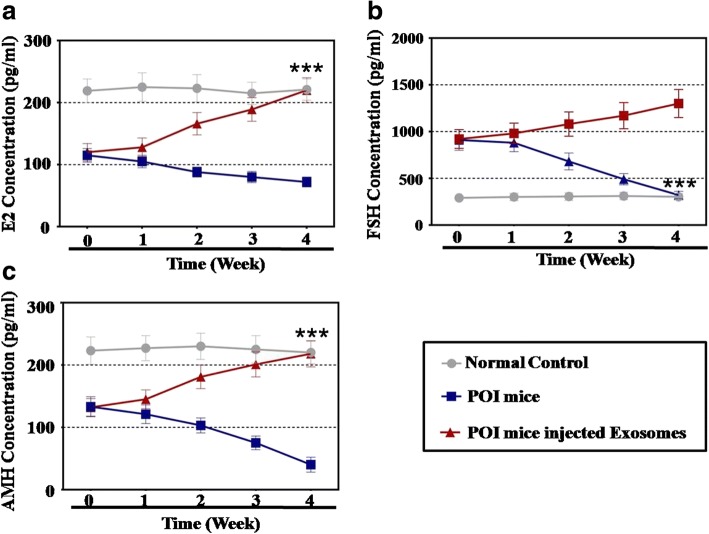
hADSC-Exos improved hormone level of POI mouse model. **a** E2 levels measured by ELISA over 4 weeks after hADSC-Exos injection. **b** FSH levels measured by ELISA over 4 weeks after hADSC-Exos injection. **c** AMH levels measured by ELISA over 4 weeks after hADSC-Exos injection. All experiments carried three times; error bars indicate SD. ***p < 0.001 (compared with POI group). E2 estradiol, FSH follicle-stimulating hormone, AMH anti-Mullerian hormone, POI premature ovarian insufficiency

The hormone level of the plasma in each group was tested after hADSC-Exos injection. In the treatment group, ELISA demonstrated that the levels of E2 (57%) and AMH (56%) were slightly rescued compared to the POI group (47% E2 and 52% AMH) and to that of the control group at week 1 (Fig. 3a, c). However, at week 4, hADSC-Exos rescued the levels of E2 (98%) and AMH (97%) to the normal levels of the control group (data with statistical significance, *p* < 0.001, compared to control group) (Fig. 3a, c). In addition, the level of FSH decreased to 267% in the treatment group compared to 327% in the POI mice group at week 1. In contrast, after hADSC-Exos treatment for 4 weeks, the level of FSH was rescued to the normal levels (106%) and was significantly different compared to the control group (data with statistical significance, *p* < 0.001, compared to control group) (Fig. 2b).

Overall, hADSC-Exos exhibited a powerful ability to restore ovarian function in a POI mouse model.

### hADSC-Exos increased the proliferation rate and inhibited the apoptosis rate of the hGCs

**Fig. 4:**
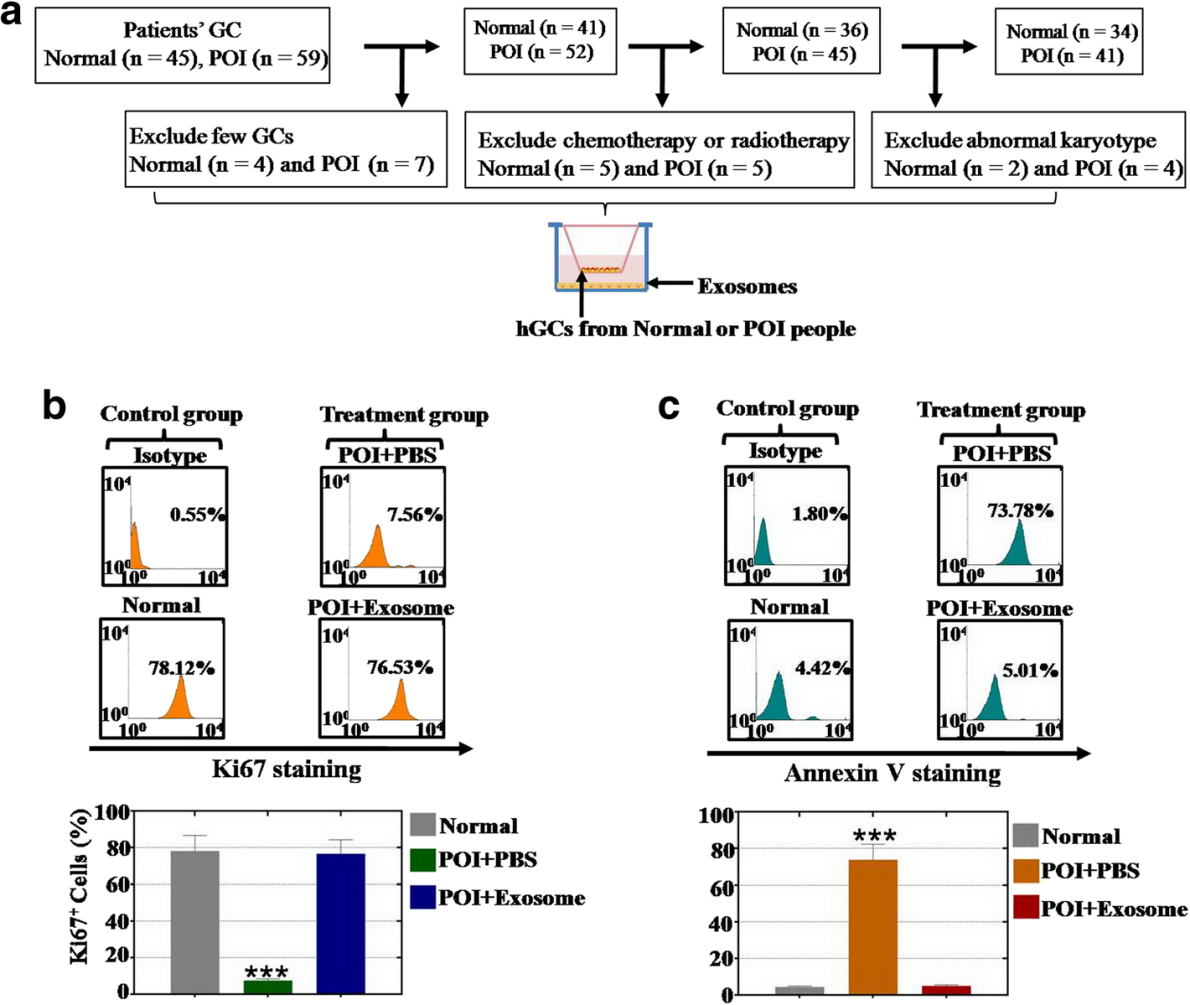
hADSC-Exos improve proliferation rate and inhibit apoptosis rate in POI hGCs. **a** Schematic overview of hGC filter procedures. **b** hADSC-Exos improve proliferation in hGCs more significantly than using PBS. **c** hADSC-Exos inhibit apoptosis in hGCs more effectively than using PBS. Error bars indicate SD. ***p < 0.001 (compared with normal group). GC granulosa cell, POI premature ovarian insufficiency, hGC human granulosa cell, PBS phosphate buffered saline

To investigate the therapeutic effects of hADSC-Exos in POI patients in the clinic, we collected hGCs from a normal group (*n* = 34) and a POI patient group (*n* = 41) in our reproductive center to examine the effects of cell proliferation after coculturing with hADSC-Exos (Fig. 4a). Annexin V antibody (cell apoptosis marker) and a ki67 antibody (cell proliferation marker) were used to estimate the effects from hADSC-Exos by a FACS analysis method. Our results showed that the proliferation rate of the POI hGCs was raised to 76% in the hADSC-Exos-injected group, which was higher than the PBS-treated group (8%) and compared to that of the normal hGCs group (79%) (Fig. 4b). Similarly, the results from the apoptosis assay indicated that hADSC-Exos decreased the rate of apoptosis to 4% in the exosome-injected group, which was lower than the PBS-treated group (74%) and compared to that of the normal hGCs group (5%) (Fig. 4c).

In summary, hADSC-Exos increased the proliferation rate and inhibited the apoptosis rate of hGCs more effectively.

### hADSC-Exos upregulated the marker expression of hGCs

**Fig. 5:**
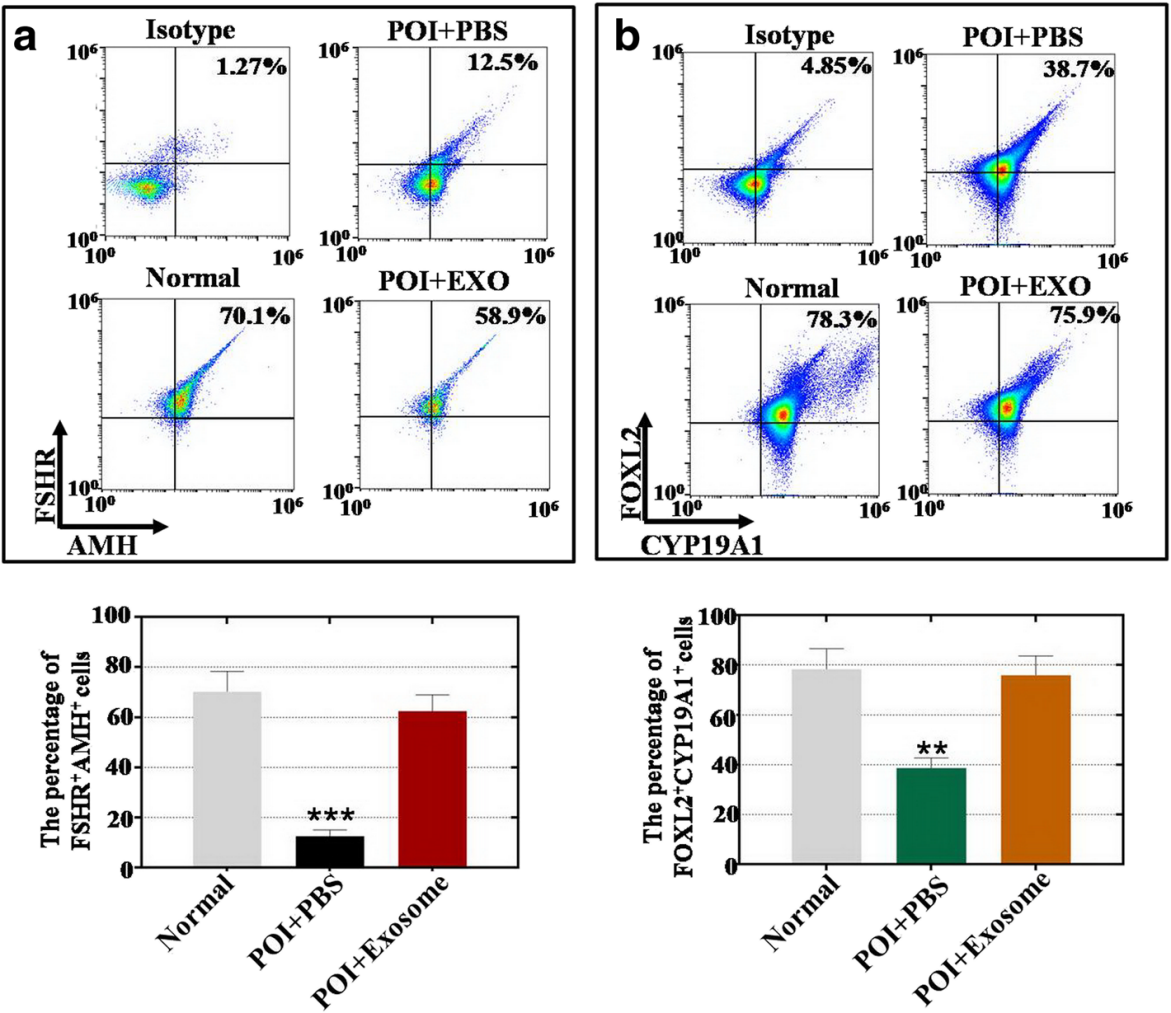
hADSC-Exos improve marker expression in POI hGCs. **a** hADSC-Exos increase number of FSHR^+^AMH^+^ hGCs. **b** hADSC-Exos increase number of FOXL2^+^CYP19A1^+^ hGCs. Error bars indicate SD. **p < 0.01, ***p < 0.001 (compared with normal group). POI premature ovarian insufficiency, PBS phosphate buffered saline, EXO exosome, FSHR follicle-stimulating hormone receptor, AMH anti-Mullerian hormone

To examine the effects of hADSC-Exos on hGC marker expression, hGCs were cocultured with hADSC-Exos for 7 days. A FACS method was employed to quantitatively assess hGC marker expression. Our results showed that hADSC-Exos significantly increased the FSHR^+^AMH^+^ cell number in the POI group to a higher level (62%) than the PBS administration group (12%, *p* < 0.001) and compared to the normal group (70%) (Fig. 5a). The FACS assay results shown in Fig. 5b demonstrate that hADSC-Exos increased the FOXL2^+^CYP19A1^+^ cell number to 76% in the POI group, significantly higher than the 39% in the PBS administration group (*p* < 0.001) and compared to that of the normal group (78%).

In total, the hADSC-Exos exhibited powerful recovery effects for POI hGCs.

### hADSC-Exos recover the effect in hGCs by regulating the SMAD pathway

**Fig. 6:**
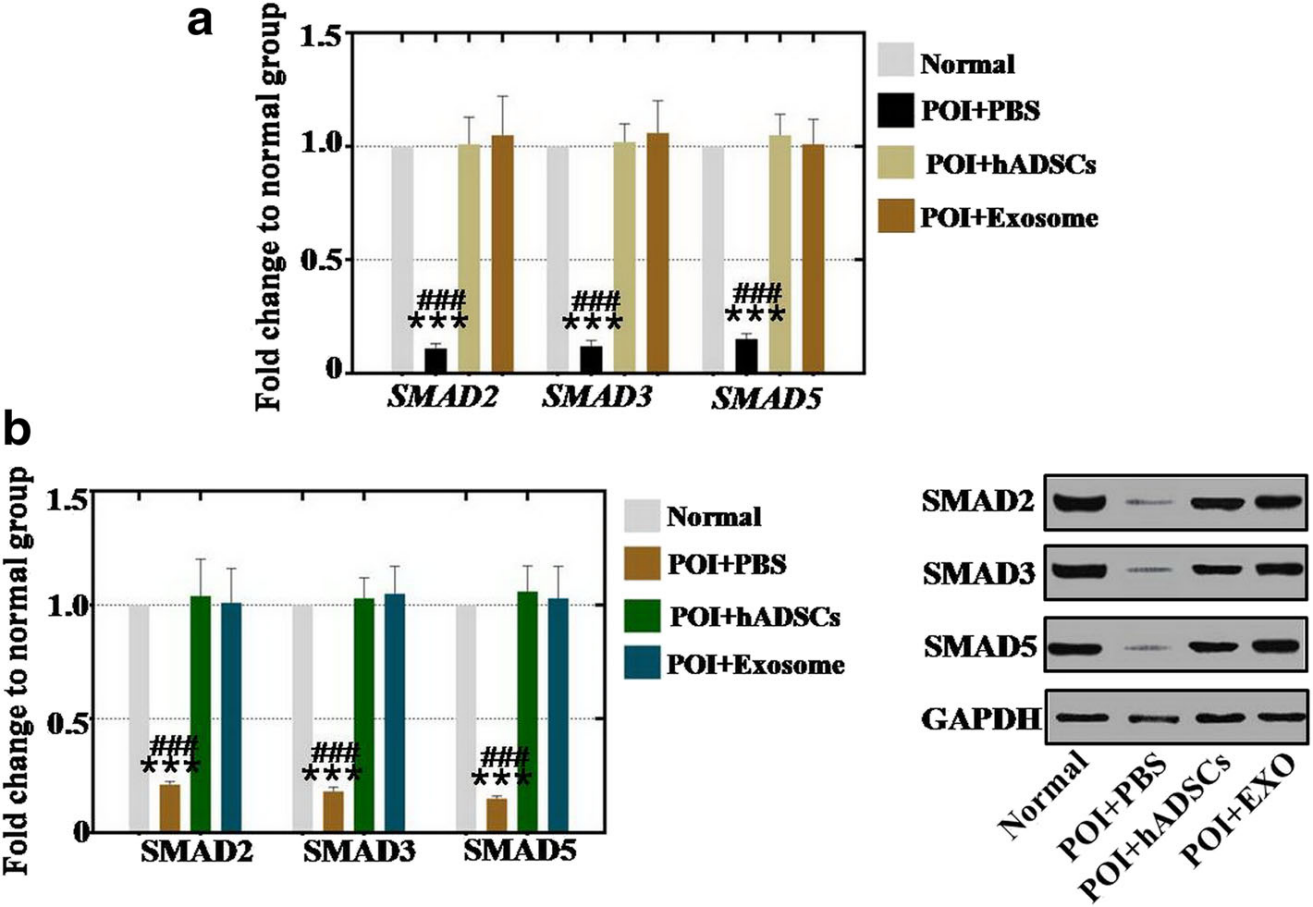
hADSC-Exos upregulated SMAD pathway in POI hGCs. **a** qPCR analysis of mRNA expression levels of SMAD2, SMAD3, and SMAD5 after hADSC-Exos coculture with POI hGCs. **b** Western blot analysis of protein expression levels of SMAD2, SMAD3, and SMAD5 after hADSC-Exos coculture with POI hGCs. Error bars indicate SD. ***p < 0.001 (compared with normal group); ###*p* < 0.001 (compared with hADSC-Exos treatment group). POI premature ovarian insufficiency, PBS phosphate buffered saline, hADSC human adipose mesenchymal stem cells, GAPDH glyceraldehyde 3-phosphate dehydrogenase, EXO exosome

To determine how treatment with hADSC-Exos improved the vitality of POI hGCs, hADSC-Exos were cocultured with hGCs for 7 days. Real-time PCR assay results showed that the gene expression levels of SMAD2, SMAD3, and SMAD5 were increased to 11%, 12%, and 15% respectively in POI hGCs compared to the normal hGC group (Fig. 6a). After treatment with hADSCs and hADSC-Exos respectively, the mRNA expression levels of SMAD2, SMAD3, and SMAD5 were significantly regulated to the normal levels (respectively 101%, 102%, and 105% in hADSC-treated group; 105%, 102%, and 101% in hADSC-Exos-treated group) compared to that in the normal hGC group (Fig. 6a).

In addition, a protein test assay also was executed in our research. Western blot assay results showed that the protein expression levels of SMAD2, SMAD3, and SMAD5 were decreased to 21%, 18%, and 15% respectively in POI hGCs compared to the normal hGC group (Fig. 6b). After treatment with hADSCs and hADSC-Exos respectively, the protein assay results indicated that hADSCs and hADSC-Exos regulated the expression of SMAD2 to 102% and 103%, of SMAD3 to 107% and 104%, and of SMAD5 to 105% and 101% respectively, compared to that in the normal hGC group (Fig. 6b).

### hADSC-Exos recover the effect in the ovary of the POI mouse model by regulating the SMAD pathway

**Fig. 7:**
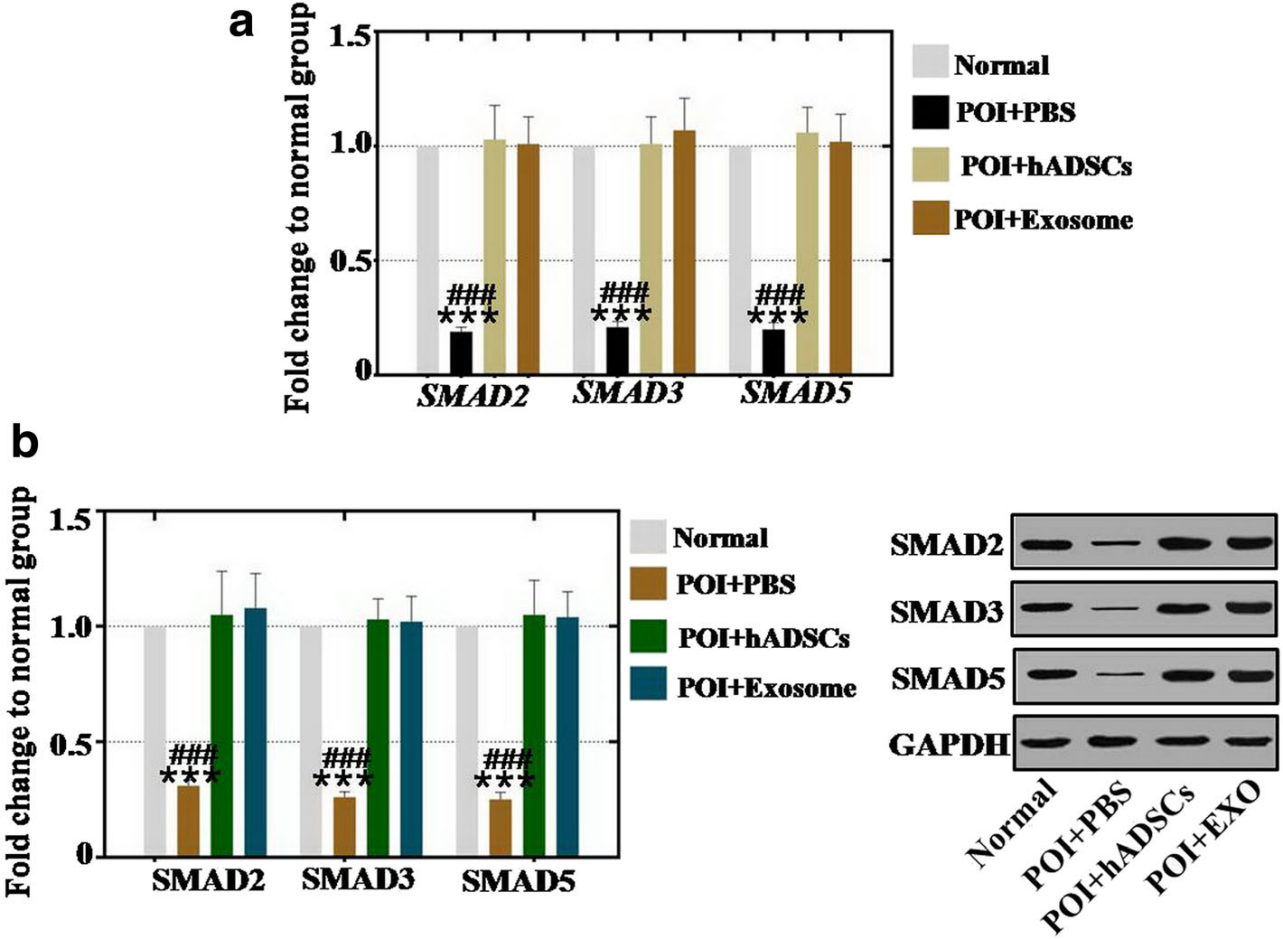
hADSC-Exos upregulated SMAD pathway in POI mouse model. **a** qPCR analysis of mRNA expression levels of SMAD2, SMAD3, and SMAD5 after hADSC-Exos injection into POI mouse model. **b** Western blot analysis of protein expression levels of SMAD2, SMAD3, and SMAD5 after injection into POI mouse model. Error bars indicate SD. ***p < 0.001 (compared with normal group); ###*p* < 0.001 (compared with hADSC-Exos treatment group). POI premature ovarian insufficiency, PBS phosphate buffered saline, hADSC human adipose mesenchymal stem cells, GAPDH glyceraldehyde 3-phosphate dehydrogenase, EXO exosome

To determine how hADSC-Exos restored ovarian function in the POI mouse model, hADSC and hADSC-Exos were injected into POI mouse ovaries several times. Real-time PCR analysis demonstrated that the gene expression levels of SMAD2, SMAD3, and SMAD5 were decreased to 19%, 21%, and 20% respectively in the POI mouse model compared to the normal mouse group (Fig. 7a). After treatment with hADSCs and hADSC-Exos respectively, the mRNA expression levels of SMAD2, SMAD3, and SMAD5 were significantly regulated to the normal levels (respectively 103%, 101%, and 106% in hADSC-treated group; 101%, 107%, and 102% in hADSC-Exos-treated group) compared to the normal mouse group (Fig. 7a).

Similar to the protein assay results, western blot analysis demonstrated that the protein expression levels of SMAD2, SMAD3, and SMAD5 were inhibited to 31%, 26%, and 25% respectively in the POI mouse model compared to the normal mouse group (Fig. 7b). After treatment with hADSCs and hADSC-Exos respectively, the protein assay results revealed that hADSCs and hADSC-Exos regulated the expression of SMAD2 to 105% and 108%, of SMAD3 to 103% and 102%, and of SMAD5 to 105% and 104% respectively, compared to that in the normal mouse group (Fig. 7b).

### hADSC-Exos repressed apoptosis genes through the SMAD pathway

**Fig. 8:**
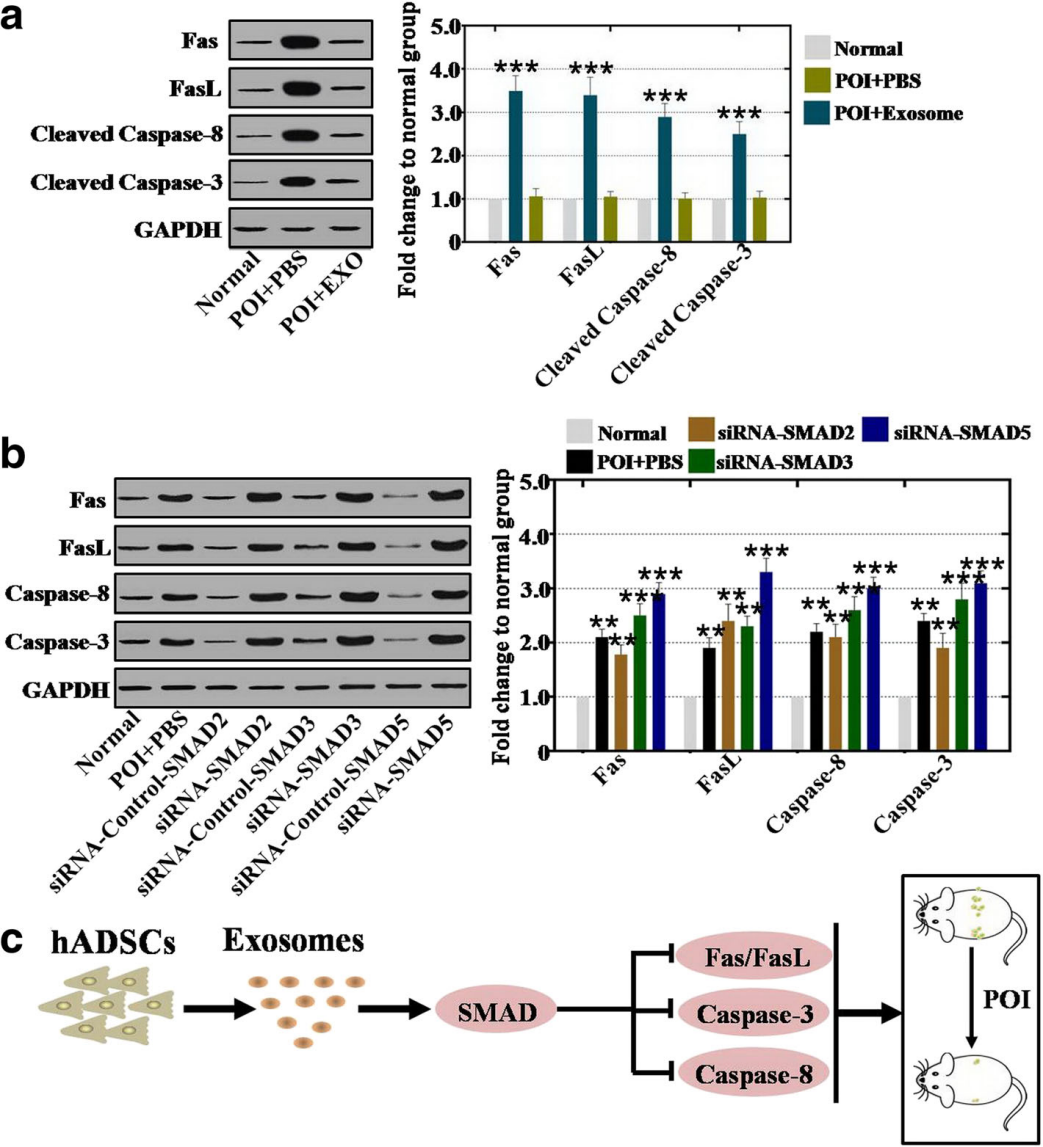
hADSC-Exos repress apoptosis genes through SMAD pathway. **a** Western blot analysis of protein expression levels of Fas, FasL, Caspase-8, and Caspase-3 after hADSC-Exos coculture with hGCs. **b** Western blot analysis of protein expression levels of Fas, FasL, Caspase-8, and Caspase-3 after SMAD2, SMAD3, and SMAD5 knockdown in normal hGCs. **c** Proposed model for exosomes derived from hADSCs improved ovaries of POI mice through regulating SMAD pathway. Error bars indicate SD. **p < 0.01, ***p < 0.001 (compared with normal group). GAPDH glyceraldehyde 3-phosphate dehydrogenase, POI premature ovarian insufficiency, PBS phosphate buffered saline, EXO exosome, siRNA small interfering RNA, hADSC human adipose mesenchymal stem cells

Furthermore, we initiated a systemic investigation into whether hADSC-Exos restored ovarian function related to apoptosis genes. Our results showed that the protein expression levels of Fas, FasL, caspase-3, and caspase-8 were elevated to 350%, 340%, 290%, and 250% respectively in the POI mouse model compared to the normal mouse group (Fig. 8a). After treatment with hADSC-Exos, the protein expression levels of Fas, FasL, caspase-3, and caspase-8 were significantly downregulated to the normal levels of 106%, 105%, 101%, and 103% respectively compared to that in the normal mouse group (Fig. 8a).

Besides, to confirm whether hADSC-Exos repressed apoptosis genes through the SMAD pathway, RNAi assay was performed in normal hGCs. Western blot assay revealed that the expression of SMAD2, SMAD3, and SMAD5 was inhibited and the protein expression levels of Fas, FasL, caspase-3, and caspase-8 were significantly upregulated to respectively 210%, 190%, 220%, and 240% in the siRNA-SMAD2 group, to respectively 250%, 230%, 260%, and 280% in the siRNA-SMAD3 group, and to respectively 290%, 330%, 300%, and 310% in the siRNA-SMAD5 group compared to the normal mouse group (Fig. 8b).

Taken together, hADSC-Exos improve the ovarian function of POI disease more effectively by regulating the SMAD pathway in vivo and in vitro.

## Discussion

A previous study showed that hADSCs have been used to restore premature ovarian failure [7]. In addition, our previous study revealed that cytokines secreted from hADSCs could resist the process of natural ovarian aging [4]. However, whether hADSCs could retard POI by releasing exosomes and how hADSC-Exos work are unknown. In a previous study, after hADSC-Exos injection, the follicle number at four stages (primordial, primary, secondary, and antral follicle stages) recovered to nearly normal levels in a POI mouse model (Fig. 2). In addition, we detected the serum index of E2, AMH, and FSH, all of which were also restored to normal levels (Fig. 3). To bridge the bench-to-bedside gap, the preclinical efficacy of hADSC-Exos for recovering the ovarian function of POI disease was evaluated by coculturing with hGCs derived from a POI patient. Earlier research also revealed that exposing a female mouse to chemotherapy downregulated the proliferation pathway and caused growing follicles to undergo apoptosis [21]. Our findings indicated that hADSC-Exos increased the proliferation rate and inhibited the apoptosis rate of hGCs to normal levels (Fig. 4). Our FACS results demonstrated that hADSC-Exos restored the marker expression levels of hGCs to normal levels (Fig. 5). Our study provides the first confirmation that hADSC-Exos have the ability to withstand POI.

Although data from several studies suggest that hADSCs play a pivotal role in wound healing, antineural aging, and angiogenesis [22, 23], few studies reveal the manner of how hADSCs work during this period. Specifically, little is known about the mechanism of how hADSCs resist POI. According to a foregoing report, study results revealed that POI caused by cytotoxic drug is associated with the SMAD signal pathway [24]. Therefore, whether hADSC-Exos play a central role in retarding POI by regulating the SMAD signal pathway needs to be clarified. At the cellular level, these study results indicated that hADSC-Exos increased the mRNA and protein expression levels of SMAD2, SMAD3, and SMAD5 to normal levels (Fig. 6). The same results were also revealed in the POI mouse model, the present results showing that hADSC-Exos reduced the mRNA and protein expression levels of SMAD2, SMAD3, and SMAD5 to normal levels (Fig. 7). Besides, SMAD knockdown elevated expression of the apoptosis genes (Fas, FasL, caspase-3, and caspase-8) significantly (Fig. 8). These current findings are consistent with the results of a previous study showing that mesenchymal stem cells recover the ovarian function of POI by regulating the SMAD pathway [25]. Several lines of evidence support this result and indicate that SMAD knockdown induced follicle loss by increasing the expression levels of apoptosis genes (Fas, FasL, caspase-3, and caspase-8) [19, 26].

## Conclusions

In summary, we investigated the interaction between hADSC-Exos and POI for the first time. The results of the present study provide insight into the mechanism by which hADSCs recover the function of POI by releasing exosomes. Furthermore, our present study revealed that the hADSC-Exos recovered the ovarian function of POI by upregulating SMAD expression. Therefore, we suggest that hADSC-Exos improve the ovarian function of POI by releasing exosomes and their effectiveness was through regulation of the SMAD pathway (Fig. 8c). This discovery has important implications for understanding the molecular mechanism by which hADSC-Exos promote the ovarian function of POI. Moreover, this discovery suggests that exosomes may serve as a novel, safer, and efficacious therapeutic schedule to resist POI and improve female reproductive health.

## Additional file

Additional file 1: **Table S1.** Designations, sequences, and sizes of real-time PCR amplicons. (DOC 39 kb)

## Abbreviations

AMH: Anti-Mullerian hormone
BMSC: Bone marrow mesenchymal stem cell
CM: Conditioned medium
CTX: Cyclophosphamide
CYP19A1: Cytochrome P450 family 19 subfamily A member 1
E2: Estradiol
ELISA: Enzyme-linked immunosorbent assay
FACS: Flow cytometry
FOXL2: Forkhead box L2
FSHR: Follicle-stimulating hormone receptor
hADSC: Human amniotic mesenchymal stem cell
HE: Hematoxylin and eosin
hGC: Human granulosa cell
POI: Premature ovarian insufficiency
SMAD: Mother against decapentaplegic-related proteins
TGF-β: Transforming growth factor beta
