# Supplementary material for: Exosomes derived from human adipose mesenchymal stem cells improve ovary function of premature ovarian insufficiency by targeting SMAD
Source: Stem Cell Res Ther. 2018 Aug 9;9:216. doi: 10.1186/s13287-018-0953-7 (PMC6085638; doi:10.1186/s13287-018-0953-7)
Supplement: Supplementary file 1 — Table S1. Designations, sequences, and sizes of real-time PCR amplicons. (DOC 39 kb) [file 13287_2018_953_MOESM1_ESM.doc]

**Table S1 Designations, sequences, and the sizes of real-time PCR amplicons**

| **Name** | **Sequence from 5'-3'** | **Size (bp)** |
| --- | --- | --- |
| SMAD2 (H) Fw | TGAAAGGGTGGGGAGCAGAATA | 136 |
| SMAD2 (H) Rev | GAGCAACGCACTGAAGGGGAT |
| SMAD3 (H) Fw | AACTGCTTCTCCCTTCTCTCTCC | 146 |
| SMAD3 (H) Rev | TTTTCATTTGCCTATGTTGGCCT |
| SMAD5 (H) Fw | CCAGTTCAGAAATTTGGCATTG | 208 |
| SMAD5 (H) Rev | CCAGTTCAGAAATTTGGCATTG |
| GAPDH (H) Fw | GAAGGTCGGAGTCAACGGATTT | 223 |
| GAPDH (H) Rev | CTGGAAGATGGTGATGGGATTTC |
| SMAD2 (M) Fw | ATGTCGTCCATCTTGCCATTC | 173 |
| SMAD2 (M) Rev | AACCGTCCTGTTTTCTTTAGCTT |
| SMAD3 (M) Fw | GCACCCTCCAATGTGATAA | 19 |
| SMAD3 (M) Rev | TTATCACATTGGAGGGTGC |
| SMAD5 (M) Fw | GAGACTGATACATACGCCTGCAGA | 81 |
| SMAD5 (M) Rev | TCACATGTCTCGATCCCAGTAGA |
| GAPDH (M) Fw | TTCCAGTATGACTCTACCCACGGCA | 137 |
| GAPDH (M) Rev | GCACCAGCATCACCCCATTTG |

**H=Human; M=Mouse.**
